# Spatiotemporal Changes in PM_2.5_ and Their Relationships with Land-Use and People in Hangzhou

**DOI:** 10.3390/ijerph15102192

**Published:** 2018-10-08

**Authors:** Li Tian, Wei Hou, Jiquan Chen, Chaonan Chen, Xiaojun Pan

**Affiliations:** 1 Qianyanzhou Ecological Research Station, Key Laboratory of Ecosystem Network Observation and Modeling, Institute of Geographic Sciences and Natural Resources Research, Chinese Academy of Sciences, Beijing 100101, China; tianli@igsnrr.ac.cn; 2 China Institute of Surveying and Mapping Science, Beijing 100830, China; 3 Department of Geography, Environment and Spatial Sciences and Center for Global Change and Earth Observations, Michigan State University, East Lansing, MI 48824, USA; jqchen@msu.edu; 4 College of Environment and Planning, Henan University, Kaifeng 475004, China; cnchen2016@163.com; 5 The Second Surveying and Mapping Institute of Zhejiang Province, Hangzhou 310012, China; zjpapxj@163.com

**Keywords:** PM_2.5_, spatial and temporal variations, land use, infants and juveniles

## Abstract

Increases in the extent and level of air pollution in Chinese cities have become a major concern of the public and burden on the government. While ample literature has focused on the status, changes and causes of air pollution (particularly on PM_2.5_ and PM_10_), significantly less is known on their effects on people. In this study we used Hangzhou, China, as our testbed to assess the direct impact of PM_2.5_ on youth populations that are more vulnerable to pollution. We used the ground monitoring data of air quality and Aerosol optical thickness (AOT) product from the Moderate Resolution Imaging Spectroradiometer (MODIS) for the spatiotemporal changes of PM_2.5_ by season in 2015. We further explored these distributions with land cover, population density and schools (kindergarten, primary school and middle school) to explore the potential impacts in seeking potential mitigation solutions. We found that the seasonal variation of PM_2.5_ concentration was winter > spring > autumn > summer. In Hangzhou, the percentage of land area exposed to PM_2.5_ > 50 µg m^−3^ accounted for 59.86% in winter, 56.62% in spring, 40.44% in autumn and 0% in summer, whereas these figures for PM_2.5_ of <35 µg m^−3^ were 70.01%, 5.28%, 5.17%, 4.16% in summer, winter, autumn and spring, respectively. As for land cover, forest experienced PM_2.5_ of 35–50 µg m^−3^ (i.e., lower than those of other cover types), likely due to the potential filtering and absorption function of the forests. More importantly, a quantitative index based on population-weighted exposure level (*pwel*) indicated that only 9.06% of the population lived in areas that met the national air quality standards. Only 1.66% (14,055) of infants and juveniles lived in areas with PM_2.5_ of <35 µg m^−3^. Considering the legacy effects of PM_2.5_ over the long-term, we highly recommend improving the monitoring systems for both air quality and people (i.e., their health conditions), with special attention paid to infants and juveniles.

## 1. Introduction

In haze days, PM_2.5_ (the particulate matter with aerodynamic diameter ≤2.5 µm) and its particle concentration account for 56.7–75.4% of the total suspended particles and >80%–90% of PM_10_ (the particles measuring ≤10 μm in aerodynamic diameter) [1]. The high rate of exposure of the youth population to PM_10_, as a consequence, was credited as one of the primary causes of decreased head and body size [2], while long-term exposure to high concentrations of PM_2.5_ was responsible for some serious health complications such as stroke, ischemic heart disease, chronic obstructive pulmonary disease, lung cancer and acute lower respiratory infection [3,4,5,6,7,8,9,10]. Currently, ambient PM_2.5_ pollution ranks the sixth among all risk factors for global premature mortalities and disability-adjusted life-years (DALYs) [11,12]. Song et al. [13] used data from the national air quality monitoring stations in 367 cities in China between 2014 and 2016 and found that the attributable mortality rate of 5–10-year-olds was 112.0 for the current year and 124.3 in 10 years. Considering the long-term legacy effects of PM_2.5_ on urban dwellers [13,14], the health risks to infants and juveniles under a severely polluted environment would increase drastically. Both the scientific community and government must take responsibility for these potential health risks and improve the overall living environment.

PM is primarily the result of rapid industrialization and motorization [15,16]. Pollution problems were widespread in European and North American cities in the 1950s and 1960s but have since become more pronounced in developing countries such as India and China [17,18]. In China, rapid economic development over the last few decades has led to worse air quality [19,20,21], with nearly no cities that meet the World Health Organization’s (WHO) Air Quality Guidelines (AQG) of PM_2.5_ < 10 µg m^−3^ [22,23,24,25]. During 2004–2012, over 93% of people in China lived in areas where PM_2.5_ exceeded China’s National Air Quality Standard for Grade II of 35 µg m^−3^ [26], due to the rapid economic development and urbanization in China. Land surface properties (e.g., roads, construction, human behavior and vegetation) can directly filter or absorb some pollutants and indirectly influence air movement through its heterogeneous urban canopies [27]. A better understanding of spatial and temporal variations of PM_2.5_, as well as its impacts on people, is urgently needed to develop effective protocols and to mitigate the impacts of PM pollution.

Some widely applied approaches for estimating PM_2.5_ and PM_10_ concentrations are based on remote-sensing data, or derived from monitoring stations. Today, we know more about the profile of aerosol particle liquidity in space but with a limited number of ground observation stations it is difficult to quantify the spatial and temporal distributions as well as the transmission characteristics of PM across a landscape [28]. Aerosol optical thickness (AOT) data collected by the Moderate Resolution Imaging Spectroradiometer (MODIS) has been proven to be a potentially useful data source on PM_2.5_ concentrations [29,30]. However, due to the dissimilarities in surface characteristics, meteorological conditions and the aerosol fine mode fraction [31,32], applications of AOT as a proxy for spatial patterns of PM have their limitations [33,34]. An alternative is to combine the dispersed monitoring data and MODIS products so that the spatiotemporal distribution of PM_2.5_ concentration can be quantified through inversion modeling.

The focal city of this study is Hangzhou—one of the central cities of the Yangtze River Delta Urban Agglomeration. We explored its atmospheric environment and found that, as a tourist city, Hangzhou’s air quality condition was not satisfactory [35,36,37,38,39,40,41,42,43,44,45,46,47,48,49]. Liu et al. [26] analyzed the atmospheric PM_2.5_ concentration and variation in Hangzhou from 2011 to 2014 and concluded that the peak occurred in 2013 (52.2 µg m^−3^) and was closely related to motor vehicle emissions and changes in meteorological conditions. Jin et al. [40] found that the PM_2.5_ particle concentration was 21.6% due to automobile fumes, 16.7% to coal burning and 12.2% to ash, soil and concrete buildings. Urban land cover and land use also had a significant impact on the spatial distribution of PM_2.5_ in urban landscapes [17,39]. With the spatial distribution of PM_2.5_ in the region, sound spatial arrangement of landscapes, combined with weather conditions, terrain formation and land use can provide meaningful solutions for mitigating the impacts of PM on people.

Our primary study objectives are to: (1) understand the spatiotemporal distribution of PM_2.5_ concentrations in Hangzhou using data from 2015; (2) examine the empirical relationships between the spatiotemporal changes of PM_2.5_ and land cover; (3) analyze the populations exposed to different levels of PM_2.5_ concentrations; and (4) analyze the distribution of students and schools (e.g., kindergarten, primary school and middle school students) living in areas of different PM_2.5_ concentrations. We aim at reducing the potential health threats of long-term exposure of infants and juveniles to PM_2.5_.

## 2. Materials and Methods

### 2.1. Study Area

**Figure 1:**
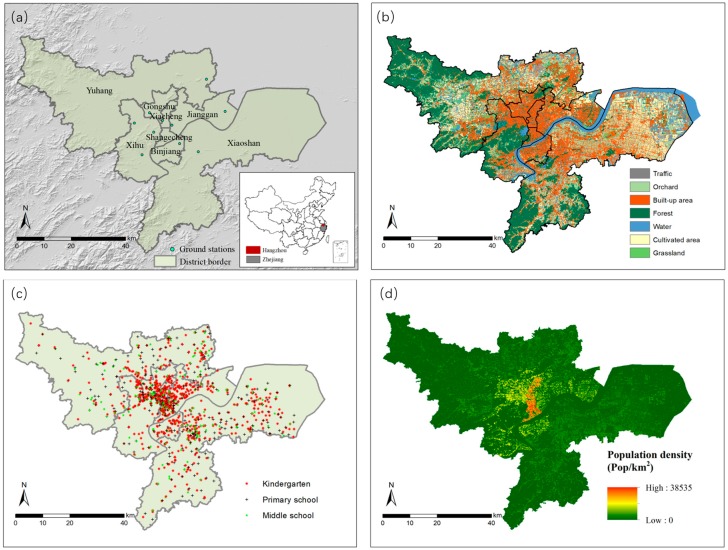
(**a**) Location of the study area and ground monitoring stations; (**b**) Land cover map of Hangzhou in 2015; (**c**) Spatial distribution of kindergarten, primary and middle schools in Hangzhou; and (**d**) Population density on 100 m × 100 m grid map.

Hangzhou city, the capital city of the Zhejiang Province, is located in southeast China. This study covers the main urban area of Hangzhou, including eight districts: Shangcheng, Xiacheng, Jianggan, Gongshu, Xihu, Binjiang, Yuhang and Xiaoshan (Figure 1a). The study area was 3376 km^2^ and the population density was 2111.96 per km^2^ in 2015 (http://www.hzfc.gov.cn/web). It experiences a humid subtropical climate with four distinct seasons and is characterized by long, hot, humid summers and chilly, cloudy winters. The average annual precipitation is 1,438 mm; rainfall is abundant during summer and relatively low during winter [35].

### 2.2. Data Sources

The MODIS has 36 spectral channels ranging from visible to infrared, providing an effective means of detecting global aerosol properties. In this study, the MOD04-3K AOT product at 3 km resolution for 2015 was acquired from the Level 1 and Atmospheric Archive and Distribution System (LAADS) (https://earthdata.nasa.gov/about/daacs/daac-laads). Geometric correction was applied to AOT images. In addition, meteorological data (e.g., wind, relative humidity from NCEP (http://dss.ucar.edu/)) were also used for calibrating AOT. Real-time hourly monitoring data of PM_2.5_ density from 10 ground stations in Hangzhou were collected from the National Environmental Monitoring Centre from January 2015 to December 2015 and were converted to daily averages to match with MODIS AOT. The land cover map was developed based on high-resolution (<1 m) aerial photos in 2015 (Figure 1b). The spatial distribution of kindergartens, primary schools, middle schools and their numbers of enrollment were collected from the Hangzhou Education Bureau (Figure 1c). The district-level population data was collected from the statistical yearbook of Hangzhou (http://tjj.hangzhou.gov.cn/tjnj/nj2017/index.htm). To create spatially continuous population distributions, we assume that the population density is highly correlated with building density. The district-level population was reallocated spatially on a standard grid map (100 m × 100 m); the proportion of build-up area in a grid was used as a weighting factor to calculate the population of a grid by ensuring that the total population of each district remains the same (Figure 1d).

### 2.3. Spatial Modeling of PM_2.5_ Distribution

**Figure 2:**
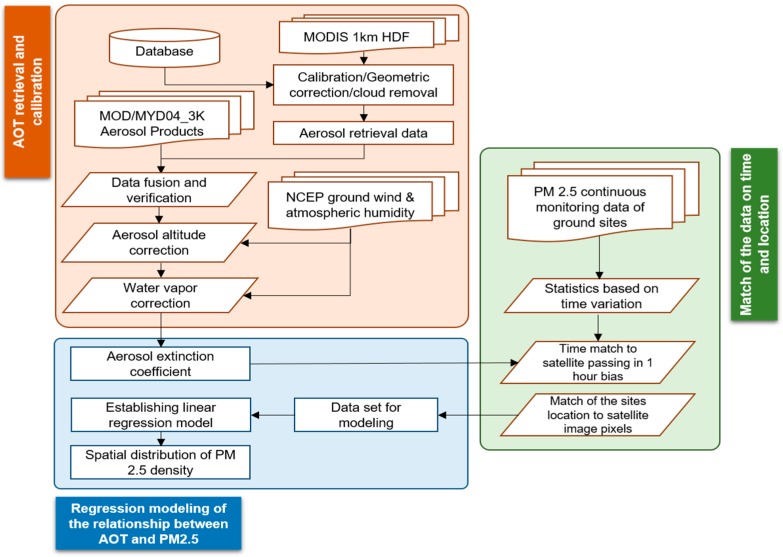
Process of modelling the relationship between aerosol optical thickness (AOT) and PM_2.5_.

Figure 2 shows the process of modeling the relationship between AOT and PM_2.5_. The three consequent steps are: AOT retrieval and calibration, match of ground monitoring data with AOT and regression modelling. The key for calibrating AOT data is correcting aerosol altitude and water vapor density. The density of aerosol particles decreases with increasing altitude because of the gravity impact. The relationship between AOT and the aerosol extinction coefficient was expressed as:

(1) $$\tau_{a}\left(\lambda \right)\approx k_{a,0}\left(\lambda \right)\times H_{A}$$

where $\tau_{a}\left(\lambda \right)$ stands for the AOT value; $k_{a,0}\left(\lambda \right)$ is the near-ground horizontal extinction coefficient, which is affected by the atmospheric water vapor content; $H_{A}$ stands for aerosol scaling height.

Aerosol scaling height is a key parameter that can be approximated by mixed-layer height. The mixed-layer height is closely related to the aerosol stability and can be calculated following the protocols of the State Bureau of Technical Supervision and the State Environmental Protection Administration [50] (Equation (2)).

(2) $$L=S\sqrt{\frac{u_{10}}{2\Omega \sin\varphi }}$$

where L stands for the mixed-layer height (m); $u_{10}$ is the wind speed at the altitude of 10 m (m s^−1^) and its maximum value is 6 m s^−1^; Ω stands for the rotational angular velocity of the earth and is assigned a value of 7.29 × 10^−5^ rad s^−1^; φ stands for geodetic latitude; S is related to the aerosol stability referring to Pasquill stability classes (see details in Reference [50]) and its corresponding values in Hanzhou can be found in Table 1.

After the corrections, the aerosol extinction coefficient can be obtained. Water vapor correction is further applied to retrieve the “dry” aerosol extinction coefficient as:

(3) $$E_{dry}=k_{a,0}\left(\lambda \right)\times \left(1-\frac{RH}{100}\right)$$

where $E_{dry}$ stands for the “dry” aerosol extinction coefficient; *RH* represents the relative humidity (%).

To establish a relationship between the ground measurements and the AOT, further statistics were applied to ensure the spatiotemporal consistency of the ground measurements with the remote sensing images. The precision of temporal match should be within ±1 h between the monitoring data and the satellite passing time. The mean value in 3 × 3 pixel cells of AOT is used for matching with the value of the monitoring location. Linear regression is applied to explore the correlation between the “dry” aerosol extinction coefficient and PM_2.5_ density (Equation (4)). Independent regression models were established by season because the climatic differences may result in different aerosol distributions.

(4) $${\mathrm{PM}}_{2.5}=a\times E_{dry}+b$$

### 2.4. Spatial Correlation between PM_2.5_ Distribution and Land Use Types

Recent studies have shown that the land cover (i.e., traffic roads) could be spatially correlated with the density of PM_2.5_ [17,51,52,53]. Incorporating this information would help to increase the model accuracy. In this study, we quantified the empirical relationship of PM_2.5_ density with different land cover types by season. Specifically, the spatial PM_2.5_ concentration was divided into three levels: <35 µg m^−3^ (non-polluted), 35–50 µg m^−3^ (intermediate) and >50 µg m^−3^ (heavy) and their proportions for each land cover type were calculated. Such results can be useful for understanding the landscape contribution and further improving PM_2.5_ predictions by including land use regression models.

### 2.5. Potential Impact of PM_2.5_ Distribution in Hangzhou

In order to estimate the impacts of PM_2.5_, several demographic data were used for calculating the proportion of the population affected by different levels of PM_2.5_. A quantitative index—population-weighted exposure level (*pwel*)—was calculated to identify the areas with potential high risk of population exposure to atmospheric particulates:

(5) $$pwel=\frac{P_{i}}{P_{total}}\times C_{i}\times 100$$

where $P_{i}$ stands for the population in grid *i* and $P_{total}$ stands for the total population in the research area; $C_{i}$ is the simulated PM_2.5_ density in grid *i*.

Additionally, the number of kindergartens, primary schools and middle schools located in different PM_2.5_ concentration zones were calculated to show the affected “key” population (i.e., infants and juveniles) in Hangzhou.

## 3. Results and Discussion

### 3.1. Relationship between AOT and the PM_2.5_ Concentration

**Figure 3:**
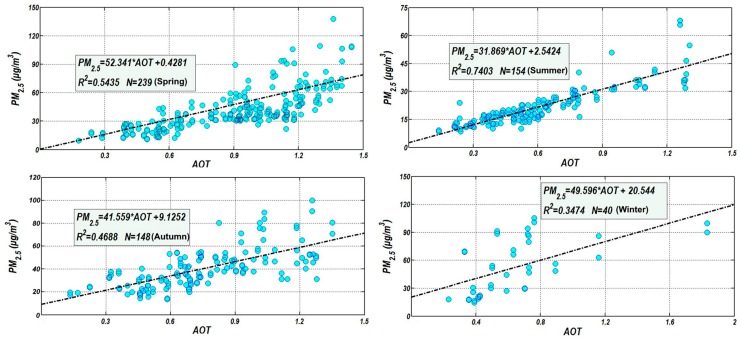
The linear regression analyses between AOT and the PM_2.5_ concentration in the four seasons in Hangzhou. (**a**) Spring; (**b**) Summer; (**c**) Autumn; (**d**) Winter.

After AOT inversion and calibration, the linear regression models were successfully established between AOT and the PM_2.5_ concentration for the four seasons (Figure 3). Model correlations varied by season, with the correlation coefficient of determination (R^2^) varying between 0.347 and 0.740. The accuracy order of the model was determined as summer > spring > autumn > winter. This seasonal difference was affected by the height of the atmospheric mixing layer (i.e., low in autumn and winter when the diffusion of air particle pollutants was low). During autumn and winter, cold waves were frequent and the resulting weather conditions led to increasing atmospheric pollutants and greater spatiotemporal variability. The model fitting accuracy decreased. Nevertheless, the appeared acceptable for all four seasons [54].

### 3.2. The Spatiotemporal Distribution in PM_2.5_

**Figure 4:**
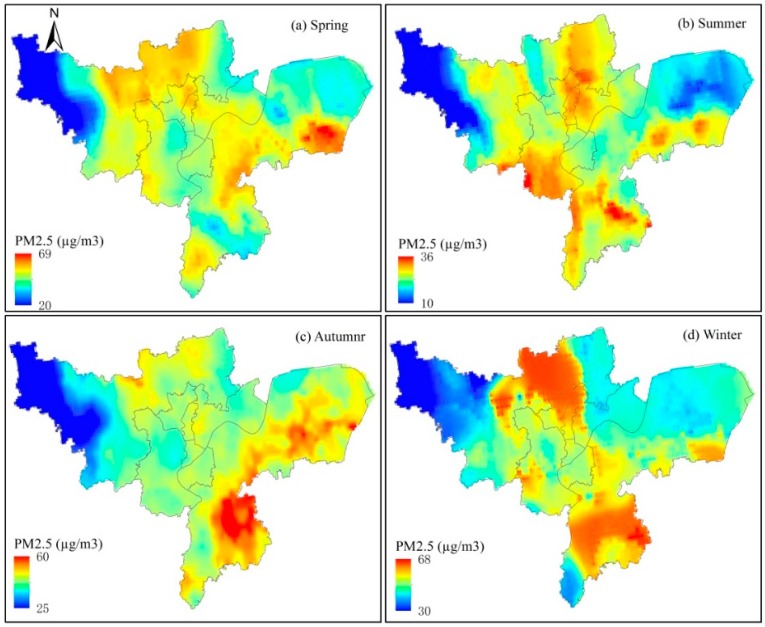
Spatial distributions of PM_2.5_ concentrations during four seasons in Hangzhou. (**a**) Spring; (**b**) Summer; (**c**) Autumn; (**d**) Winter.

The annual average of PM_2.5_ concentrations was 43 µg m^−3^ (std = 5.28), indicating that Hangzhou’s regional air quality was better than half of China’s cities (53.0 µg m^−3^) in 2015 [13,27]. Following China’s peak PM value in 2013, the PM_2.5_ concentration showed a significant decreasing trend. In Hangzhou, the PM_2.5_ concentration was 52.2 µg m^−3^ in 2013 [42]. However, this level is far from China’s National Air Quality Standard for Grade II limit of 35 µg m^−3^ [22]. Among the 366 cities in China, over 80% did not reach the standard of Grade II [55]. As for the spatial distribution of the annual average, PM_2.5_ was mainly concentrated in Gongchu, Shangcheng, Xiacheng and parts of Xihu, Yuhang and Xiaoshan. The mean PM_2.5_ concentration was 50.27 µg m^−3^ (std = 7.32) in spring, 24.87 µg m^−3^ (std = 4.40) in summer, 43.63 µg m^−3^ (std = 5.66) in autumn and 53.19 µg m^−3^ (std = 6.92) in winter (Figure 4a–d). The lowest values were found in the northwest mountainous areas (Figure 4). Among the seasons, the concentration was winter > spring > autumn > summer. The seasonal characteristics of PM_2.5_ concentration were consistent with the ground observations in Hangzhou. During winter, air pollution remained as a serious issue that severely affected people. The administrative department continued to struggle to find efficient ways to reduce the pollution level [56,57,58,59].

Among the eight districts, the Xiacheng district showed three seasons with the highest mean values (Figure 4, Table 2), with 54.10 µg m^−3^ in spring, 29.73 µg m^−3^ in summer and 45.27 µg m^−3^ in autumn. The Gongshu district had the highest value during winter (61.08 µg m^−3^). The lowest value of PM_2.5_ concentrations for all four seasons appeared in the Yuhang district, with 47.65 µg m^−3^ in spring, 23.31 µg m^−3^ in summer, 39.41 µg m^−3^ in autumn and 54.63 µg m^−3^ in winter. Interestingly, the Xiaoshan district showed the highest value in all four seasons, with 68.54 µg m^−3^ in spring, 35.95 µg m^−3^ in summer, 59.63 µg m^−3^ in autumn and 67.97 µg m^−3^ in winter (Table 2). Additionally, there appeared multiple “hot spots” in all four seasons (Figure 4).

**Figure 5:**
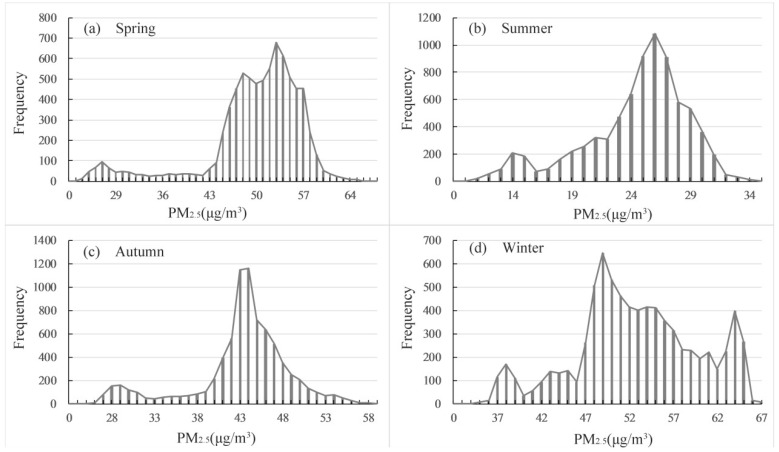
Histograms depicting PM_2.5_ concentration statistics for four seasons in Hangzhou. (**a**) Spring; (**b**) Summer; (**c**) Autumn; (**d**) Winter.

The histogram statistics of PM_2.5_ for the four seasons were also calculated. The distributions of PM_2.5_ in spring and autumn were relatively narrow, presenting a typical single peak distribution (Figure 5a,c). However, winter and summer showed dispersed values for PM_2.5_; these values were especially complex for winter, where there were multiple peaks (Figure 5b,d). This complexity was likely due to winter’s mixed atmospheric layer height being low and not conductive to the diffusion of atmospheric particle pollutants. In addition, cold waves frequently changed the weather conditions, which subsequently led to an increase in the spatiotemporal variability of atmospheric pollution and resulted in the regularity of distribution being less significant than that of spring and autumn [15,16]. Even in northern China, the distribution of PM_2.5_ was complex and contained multiple peaks during the winter [57,60].

### 3.3. Correlation Analysis between Land Use and the Spatial Distribution of PM_2.5_ Concentration

Of the seven land cover types in the study area, the landscape was composed of 26.86% (905.84 km^2^) built-up area, 12.87% (434.22 km^2^) water, 4.94% (166.23 km^2^) grassland, 26.31% (887.42 km^2^) forest, 11.95% (403.07 km^2^), cultivated land, 5.38% (181.53 km^2^), roads, 11.67% (393.91 km^2^) and orchard land (Figure 1b). We delineated portions of the seven land cover types by three classes of PM_2.5_ concentrations (Table 3). In spring, 4.16% of the land surface in Hangzhou experienced PM_2.5_ of <35 µg m^−3^. Meanwhile, 39.23% and 56.62% of the land surface was experiencing air conditions of PM_2.5_ between 35–50 µg m^−3^ and PM_2.5_ of >50 µg m^−3^, respectively. In summer, the air quality was better, as 70.01% of the land surface was exposed to PM_2.5_ of <35 µg m^−3^ and no area accounted for PM_2.5_ of >50 µg m^−3^. In autumn, only 5.17% of the land surface experienced PM_2.5_ of <35 µg m^−3^ and 40.44% of the land surface was exposed to PM_2.5_ of >50 µg m^−3^. The air pollution in winter was more severe, with 59.86% of the land surface under PM_2.5_ of >50 µg m^−3^. Overall, the PM_2.5_ concentrations in the winter and spring seasons were higher than those of the other seasons and showed multiple peaks (Figure 5). Hangzhou’s meteorological conditions in these seasons were not conducive to the emission of air pollutants [37,44,49].

In regard to the land cover types, the forests in Hangzhou were distributed mainly around the area where the PM_2.5_ concentration was 35–50 µg m^−3^. Within PM_2.5_ of <35 µg m^−3^, forests occupied the highest proportion of the land surface (Table 3). This may due to the filtering/abortion function as particulate air pollutants move through the forest landscape [17,61,62]. Janhäll [27] has advocated that increasing the vegetation barriers should help absorb and filter the particulate air pollution. For PM_2.5_ of >50 µg m^−3^, built-up area showed the largest area proportion in spring, autumn and winter (Table 3). This result again highlighted the trend of ‘more human activities, more air pollution sources’ [26].

### 3.4. Population Group Exposure under the Roof of the PM_2.5_

Based on the annual mean PM_2.5_ concentration and its spatial distribution in Hangzhou, the population-weighted exposure level (*pwel*) showed the risk level of populations exposed to different concentrations of PM_2.5_. We found 249.18 Pop km^−2^ (±746.53) of the population live in PM_2.5_ of <35 µg m^−3^, covering 266.29 km^2^; for PM_2.5_ of 35–50 µg m^−3^, the population density was 1521.60 Pop km^−2^ (±3584.08) in 1483.99 km^2^; for PM_2.5_ of >50 µg m^−3^, the population density was 1582.66 Pop km^−2^ (±3124.79) in 1188.18 km^2^ (Figure 1d). Clearly, most people reside in high PM_2.5_ concentration areas. On the other hand, gaseous and particulate pollutants were also exposed due to human activity. Pollution from human activities has severely contributed to the health impacts on people over a long period of time [63]. Considering infants and juveniles attending school, younger individuals are less resistant to disease and daily exposure to high PM_2.5_ concentrations can cause both current and future health problems.

In the study area, the number of kindergartens was 623, with 239,459 infants. The number of primary schools was 265, facilitating 389,260 students. The number of middle schools was 123, with 217,959 students. By the different PM_2.5_ concentration levels, 294 kindergarten students were under PM_2.5_ of >50 µg m^−3^, 325 under 35–50 µg m^−3^ and only four under <35 µg m^−3^. Seven primary schools were under <35 µg m^−3^, 147 under 35–50 µg m^−3^ and 111 under >50 µg m^−3^. Two middle schools experienced PM_2.5_ of <5 µg m^−3^, 123 middle schools in 35–50 µg m^−3^ and 71 middle schools in >50 µg m^−3^ (Table 4). These results indicated that at each of the aforementioned educational levels, only 1.66% (14,055) of infants and juveniles lived in an environment that met China’s National Air Quality Standard for Grade II. This number fell far below the national mean level [13,26,64]. In addition, 41.97% (355,333) of infants and juveniles lived in a heavily polluted environment (PM_2.5_ > 50 µg m^−3^) and 56.49% (478,257) of infants and juveniles lived in an intermediately polluted environment (PM_2.5_ of 35–50 µg m^−3^) (Table 4). Although we only generated statistics for the number of infants and juveniles, the families and schools near the residential areas experienced a similar atmospheric environment. Although children’s disease attributed to PM_2.5_ exposure has not been well studied, other studies have showed that China’s leading mortality causes (e.g., stroke, IHD, LC and COPD) could be attributed to PM_2.5_ exposure to some extent [35,64]. Considering the legacy effects on human health from long-term PM_2.5_ exposure, it is necessary to track the health status of infants and juveniles from birth until they have entered into adulthood. By doing so, we might reduce the harms of PM_2.5_ on people.

## 4. Conclusions

We used a combination of the dispersed monitoring ground data, land cover data and MODIS remote-sensing AOT to model the distribution of PM_2.5_ concentrations and to analyze its effects on residents, with a particular focus on infants and juveniles attending schools in Hangzhou in 2015. First, the seasonal variation in PM_2.5_ concentration was winter > spring > autumn > summer. For the eight main urban districts, the highest PM_2.5_ concentrations in spring, summer and autumn were located in the Xiacheng district and the lowest value was located in the Yuhang district. However, in winter, the highest value was found in the Gongshu district and the lowest value in the Yuhang district. In addition, the lowest value for all four seasons appeared in the Yuhang district, where there is abundant vegetation and a low population density. Secondly, for the different land cover types, we found that in winter and spring, 59.86% and 56.62% of the land area was exposed to PM_2.5_ concentrations of >50 µg m^−3^, while the built-up area occupied 20.65% in winter and 19.72% in spring. In autumn, 54.38% of the land area was exposed PM_2.5_ 35–50 µg m^−3^ and forest occupied the largest proportion (15.49%). In the summer, the air particulate content was the lowest, with 70.01% of the land surface area exposed to PM_2.5_ of <35 µg m^−3^ and the forests accounted for 23.39%. Finally, based on the spatial distribution of different classes of PM_2.5_ concentrations, only 9.06% of the population lived in an environment that met the national air quality standards. For infants and juveniles, only 1.66% (14,055) lived in areas of PM_2.5_ of <35 µg m^−3^; 56.49% of infants and juveniles (478,257) lived in an intermediately polluted environment (PM_2.5_ of 35–50 µg m^−3^) and 41.97% (355,333) lived in a heavily polluted environment (PM_2.5_ > 50 µg m^−3^) in Hanzhou. We estimated site-specific annual PM_2.5_ concentrations. Most infants and juveniles currently live in an atmospherically polluted environment not only in Hangzhou but also in most cities in China. We believe that air quality modelling and cost-benefit analyses of emission reduction scenarios and corresponding health benefits play key roles in meeting the site-specific annual PM_2.5_ concentration goals. Actions must be taken and attention must be paid in order to safeguard the future of the country.

## Figures and Tables

**Table 1:** Mixed layer parameter reference table.

Level of Stability	Extremely Unstable	Moderately Unstable	Slightly Unstable	Neutral	Moderately Stable	Stable
S	0.056	0.029	0.020	0.012	1.660	0.700

**Table 2:** PM_2.5_ concentrations in the eight districts of Hangzhou by season in 2015, including maximum, minimum, mean and standard deviation values.

		Shangcheng	Xiacheng	Jianggan	Xihu	Gongshu	Binjiang	Yuhang	Xiaoshan
Spring	Max	51.69	58.70	57.11	58.97	59.49	54.13	61.18	68.54
	Min	47.67	49.04	45.38	46.53	48.84	48.26	23.48	41.07
	Mean	50.32	54.10	52.21	51.94	53.87	51.26	47.65	51.80
	Std	0.94	3.06	2.77	2.64	2.54	0.92	10.29	4.61
Summer	Max	28.14	31.95	32.78	34.94	31.76	29.30	33.03	35.95
	Min	24.28	26.79	22.37	23.89	23.40	23.34	10.84	16.69
	Mean	25.87	29.73	27.11	28.43	27.38	25.49	23.31	24.96
	Std	1.02	1.13	2.29	2.27	2.26	1.33	5.36	3.50
Autumn	Max	45.88	46.51	47.77	45.85	45.63	46.06	50.95	59.63
	Min	41.76	43.76	41.09	40.16	42.51	43.41	24.83	40.80
	Mean	44.12	45.27	44.14	43.35	43.96	44.49	39.41	47.33
	Std	1.03	0.67	1.32	1.18	0.68	0.57	6.30	3.40
Winter	Max	57.09	64.74	64.43	65.57	65.42	59.97	66.47	67.97
	Min	53.03	54.21	46.90	48.26	53.83	51.70	44.06	44.06
	Mean	54.67	60.19	54.67	55.14	61.08	54.82	54.63	54.63
	Std	1.06	2.86	4.49	2.61	3.86	1.61	8.56	5.60

**Table 3:** The percentage area of the different land use types to the total study area percentage (%) by the three levels of PM_2.5_ concentration in the four seasons.

Land Use Type	PM_2.5_ Class (µg m^−3^)	Spring (%)	Summer (%)	Autumn (%)	Winter (%)
Grassland	<35	0.07	4.59	0.11	0.10
Cultivated area	<35	0.13	11.52	0.25	0.16
Built-up area	<35	0.17	2.51	0.26	0.25
Traffic area	<35	0.05	4.92	0.07	0.07
Forest	<35	3.49	23.39	3.93	4.35
Water	<35	0.03	12.21	0.10	0.05
Orchard	<35	0.22	10.87	0.45	0.30
Grassland	35–50	1.84	0.40	3.03	1.57
Cultivated area	35–50	5.27	0.59	6.35	5.23
Built-up area	35–50	6.74	24.12	14.35	5.73
Traffic area	35–50	1.41	0.55	3.11	1.38
Forest	35–50	13.76	2.82	15.49	14.81
Water	35–50	6.32	0.60	7.39	3.12
Orchard	35–50	3.89	0.92	4.66	3.00
Grassland	>50	3.09	0	1.84	3.33
Cultivated area	>50	6.71	0	5.51	6.71
Built-up area	>50	19.72	0	12.02	20.65
Traffic area	>50	4.02	0	2.29	4.02
Forest	>50	8.95	0	6.78	7.03
Water	>50	6.46	0	5.32	9.64
Orchard	>50	7.67	0	6.68	8.48

**Table 4:** The number of Kindergarten, Primary School and the Middle Schools by the three classes of annual PM_2.5_ concentration and the population density.

PM_2.5_ (µg m^−3^)	Kindergarten	Primary School	Middle School
<35	4	7	2
35–50	325	147	123
>50	294	111	71
Total School	623	265	196
Total population	239,459	389,260	217,959
Mean (Pop/School)	384	1469	1118
